# Hepatopancreas immune response during molt cycle in the mud crab, *Scylla paramamosain*

**DOI:** 10.1038/s41598-020-70139-2

**Published:** 2020-08-04

**Authors:** Zhanning Xu, An Liu, Shengkang Li, Guizhong Wang, Haihui Ye

**Affiliations:** 1 College of Ocean and Earth Sciences, Xiamen University, Xiamen, 361102 China; 2 Guangdong Provincial Key Laboratory of Marine Biology, Shantou University, Shantou, 515063 China

**Keywords:** Biological techniques, Molecular biology

## Abstract

Molt is a critical developmental process in crustaceans. Recent studies have shown that the hepatopancreas is an important source of innate immune molecules, yet hepatopancreatic patterns of gene expression during the molt cycle which may underlie changes in immune mechanism are unknown. In this study, we performed Illumina sequencing for the hepatopancreas of the mud crab, *Scylla paramamosain* during molt cycle (pre-molt stage, post-molt stage, and inter-molt stage). A total of 44.55 Gb high-quality reads were obtained from the normalized cDNA of hepatopancreas. A total of 70,591 transcripts were assembled; 55,167 unigenes were identified. Transcriptomic comparison revealed 948 differentially expressed genes (DEGs) in the hepatopancreas from the three molt stages. We found that genes associated with immune response patterns changed in expression during the molt cycle. Antimicrobial peptide genes, inflammatory response genes, Toll signaling pathway factors, the phenoloxidase system, antioxidant enzymes, metal-binding proteins and other immune related genes are significantly up-regulated at the post-molt stage and inter-molt stage compared with the pre-molt stage, respectively. These genes are either not expressed or are expressed at low levels at the pre-molt stage. To our knowledge, this is the first systematic transcriptome analysis of genes capable of mobilizing a hepatopancreas immune response during the molt cycle in crustaceans, and this study will contribute to a better understanding of the hepatopancreas immune system and mud crab prophylactic immune mechanisms at the post-molt stage.

## Introduction

In crustaceans, molt is an essential characteristic of the organism's development; in order to grow, the animal must shed the old exoskeleton and construct a new one^1–4^. The crustacean molt cycle affects the status of a number of physiological processes, including the interaction with environmental stressors and disease agents^5,6^. In crustaceans, several researchers have described studies of molt susceptibility to pathogenic infections. Corteel et al.^7^ reported that the Pacific whiteleg shrimp, *Litopenaeus vannamei* at post-molt stage is more susceptible to white spot syndrome virus (WSSV) infection than at pre-molt stage. Tumburu et al.^8^ have also shown that pesticide-virus interactions lead to increasingly higher susceptibility to acute toxicity at post-molt stage in *L. vannamei*. These studies indicate that crustaceans display susceptibility to pathogenic infections that vary during the molt cycle.

The hepatopancreas is an important organ involved in the process of crustacean molting, and plays a vital role in energy storage and breakdown, nutrient accumulation, and carbohydrate and lipid metabolism^9^. Growth and developmental metabolism related genes that are involved in the formation of the molt have been isolated in crustaceans. However, despite extensive research, the changes in hepatopancreas immune response during the molt cycle in crustaceans still remain poorly understood. Recent findings show that the hepatopancreas is an integrated organ of immunity and metabolism, which is an important source of innate immune molecules in crustaceans^10–13^, while nutrition and metabolic regulation may have an impact on the efficiency of the immune response in the hepatopancreas^14^. At post-molt stage, in light of the soft body, lack of exoskeleton protection, and susceptibility to various pathogens, crustaceans may mobilize the body's immune defense^4^.

Mud crabs (*Scylla* species) are found throughout tropical and warm temperate zones in the Indo-Pacific, and *S. paramamosain* is a commercially important marine fishery species in the Southeast coast of China^15^. Transcriptomics is an emerging discipline, which can now be used to examine patterns of gene transcription relative to crustacean immunity, growth and molting^12,14,16^. In this study, we used RNA-Seq to investigate the immune responses in the hepatopancreas at three molt stages in the mud crab, *S. paramamosain* to understand the mechanism of immune defense during the crustacean molt cycle.

## Results

### Sequencing and de novo assembly of hepatopancreas transcriptome

After sequencing, quality trimming, and adapter clipping, a total of 176,833,411 paired-end reads were obtained from 9 samples at the three molt stages (post-molt stage, inter-molt stage and pre-molt stage) and used for de novo assembly. We obtained 70,591 transcripts after assembly, and 55,167 unigenes were identified (Table 1).

**Table 1:** Summary of sequence analysis in *S. paramamosain* hepatopancreas.

Description	Number
**Clean sequencing reads**	
Clean reads	176,833,411
Total nucleotides (bp)	4.5 × 10^10^
Percentage of GC content (%)	50.88%
**Alignment statistics**	
Mapped reads	134,832,741
Mapped ratio	76.25%
**Assembly statistics**	
The number of transcript	70,591
The number of unigene	55,167
**Annotation**	
COG	7,742
GO	8,260
KEGG	10,495
KOG	15,436
Pfam	19,147
Swissprot	21,846
NCBI-NR	21,764

### Identification of differentially expressed genes (DEGs)

In this study, the analysis of hepatopancreas transcriptome data revealed that 948 DEGs were annotated successfully across the three molting stages (Table 2). We found that the DEGs between the post-molt stage and inter-molt stage were relatively few, with only 6 genes identified. The DEGs between inter-molt and pre-molt stage, or between post-molt and pre-molt stage were substantially more, being 789 and 153 genes, respectively.

**Table 2:** Statistics of DEGs in the hepatopancreas transcriptome of *S. paramamosain.*

All genes		Significant DEGs (Padj < 0.01)	
Cond.1	Cond.2	Up-regulation (Cond.2 > Cond.1)	Down-regulation (Cond.2 < Cond.1)
Post-molt stage	Inter-molt stage	5	1
Inter-molt stage	Pre-molt stage	188	601
Pre-molt stage	Post-molt stage	142	11

*Padj* *p* value adjusted for multiple testing using Benjamini–Hochberg.

### Functional classification of DEGs of hepatopancreas

**Figure 1:**
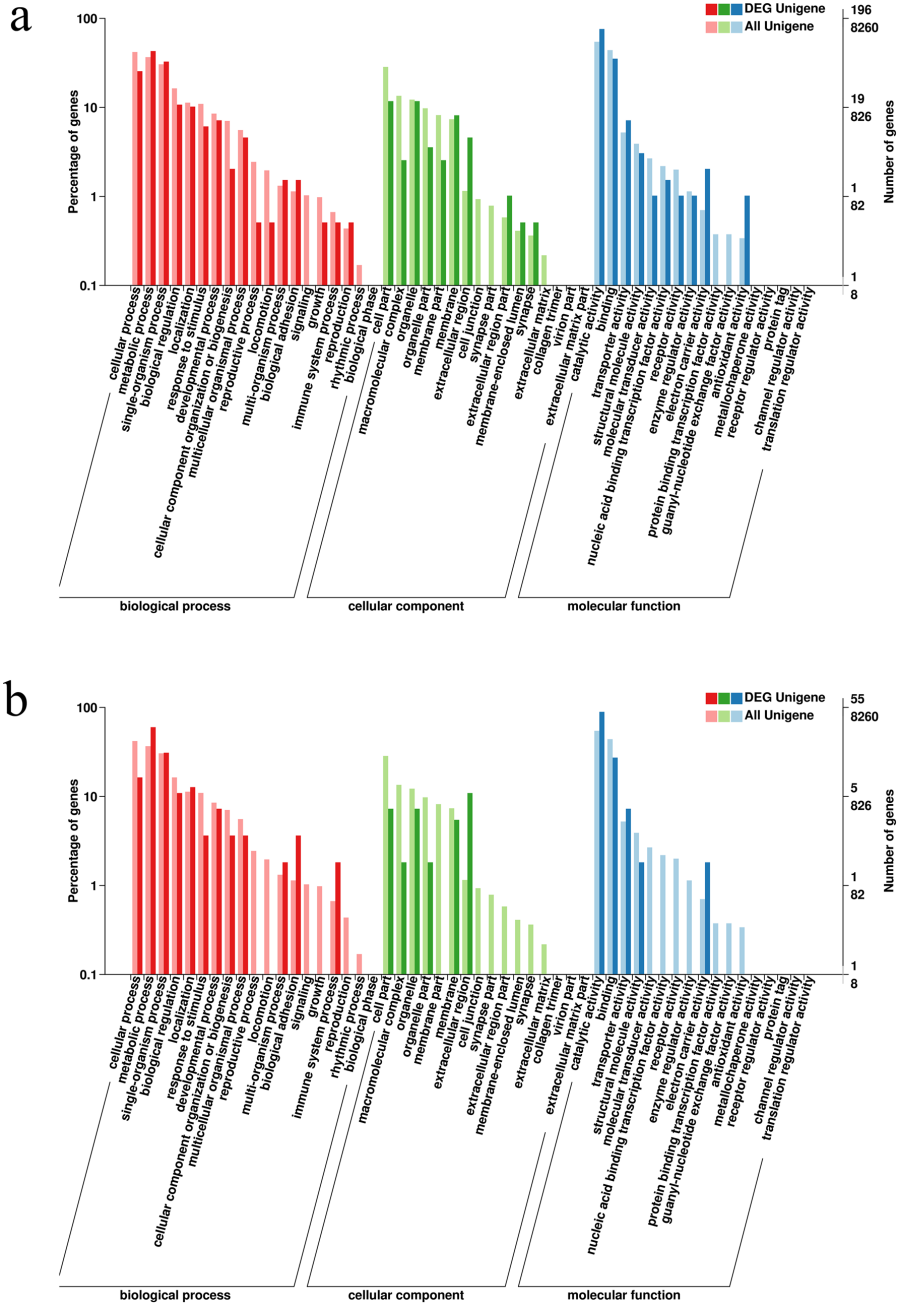
Distribution of Gene Ontology (GO) functional categories for *S. paramamosain* hepatopancreas transcriptome. (**a**) Functional classification of DEG unigenes compared between inter-molt stage and pre-molt stage in the GO database; (**b**) Functional classification of DEG unigenes compared between pre-molt stage and post-molt stage in the GO database; GO functional categories is mined here from https://www.geneontology.org. Note: the x-axis indicates the secondary classification of the GO database; the y-axis on the left side indicates the ratio of the number of genes annotated to this GO classification to all genes (the denominator of ‘All Unigene’ is all unigenes, and the denominator of ‘DEG Unigene’ is all DEG unigenes). The y-axis on the right side indicates the number of genes annotated to this GO entry. The number above represents the number of DEG unigenes, and the number below represents the number of all unigenes.

Assembled non-redundant unigenes were subjected to Gene Ontology (GO)^17^, Clusters of Orthologous Groups (COG)^18^ and Kyoto Encyclopedia of Genes and Genomes (KEGG)^19^ databases for blast searching^20^. GO annotation output showed that the unigenes could be assigned to three parts: biological processes, cellular components, and molecular functions. GO function classification was performed with DEGs. In the hepatopancreas, compared post-molt stage and inter-molt stage, the three major functional categories (biological process, cellular component, and molecular function) were not found in the GO terms associated with significant DEGs. When inter-molt stage and pre-molt stage were compared, the DEGs were assigned to 36 terms, of which dominant subcategories included single-organism process (64 genes) and metabolic process (84 genes) in the category of biological process, organelle (23 genes) and cell part (23 genes) in cellular component, and catalytic activity (149 genes) in molecular function, respectively (Fig. 1a). In the comparison of pre-molt stage and post-molt stage, metabolic process (33 genes) was the top abundant terms in biological process. While, extracellular region (6 genes) and catalytic activity (49 genes) were the top terms in the cellular component and molecular function, respectively (Fig. 1b) (Supplementary data 1).

**Figure 2:**
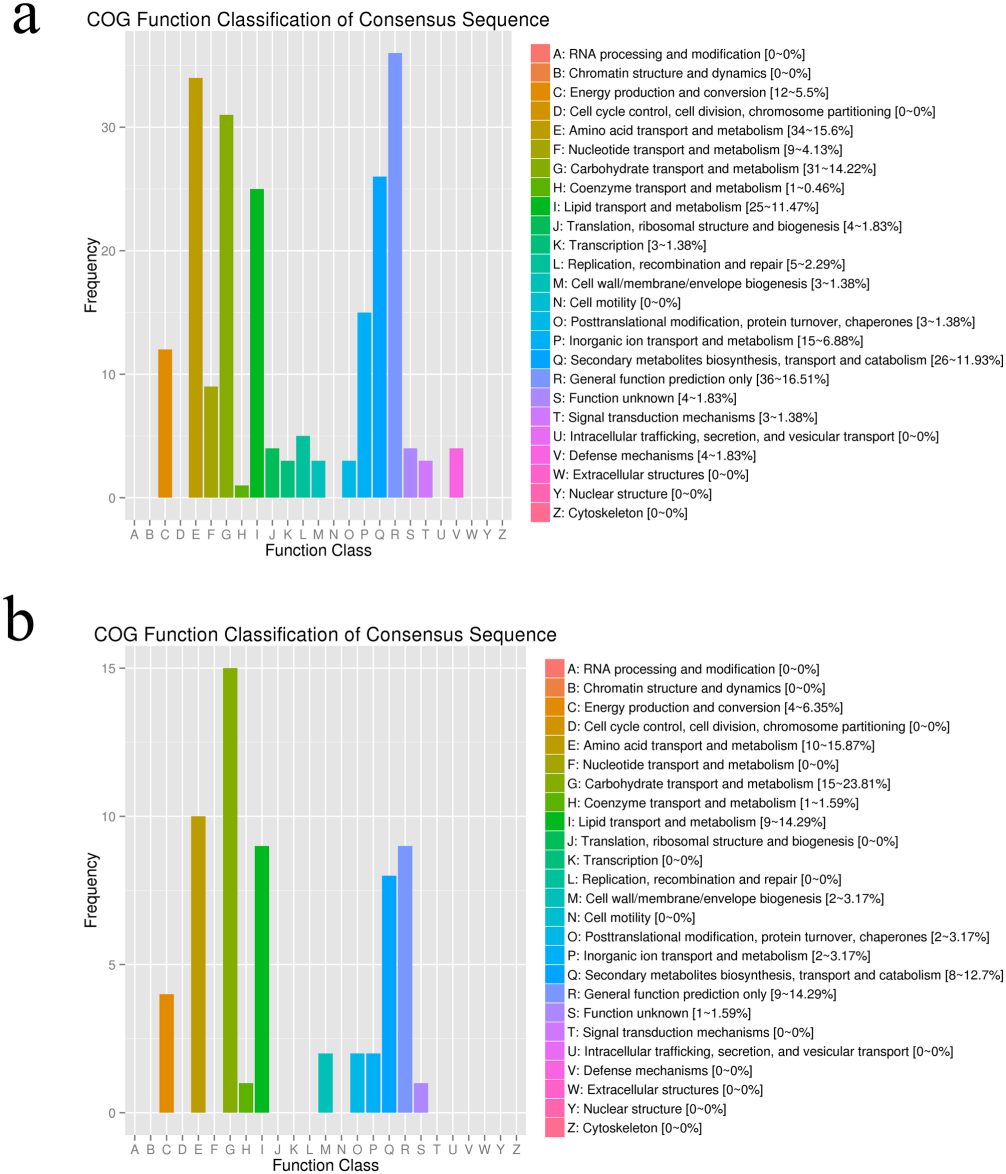
COG classification of putative proteins for *S. paramamosain* hepatopancreas transcriptome. (**a**) COG annotations of the DEGs at inter-molt stage and pre-molt stage. (**b**) COG annotations of the DEGs at pre-molt stage and post-molt stage. COG classification is mined here from https://www.ncbi.nlm.nih.gov/COG.

To classify orthologous gene products, COG function classification was performed with significant DEGs. In the hepatopancreas, DEGs at post-molt stage and inter-molt stage were not found in the COG annotations. In the comparison of inter-molt stage and pre-molt stage, the cluster of “general function prediction only” (36, 16.51%) represented the largest group, followed by “amino acid transport and metabolism” (34, 15.6%), “carbohydrate transport and metabolism” (31, 14.22%), “Secondary metabolism biosynthesis, transport and catabolism” (26, 11.9%), “lipid transport and metabolism” (25, 11.47%) and “inorganic ion transport and metabolism” (15, 6.88%), whereas “coenzyme transport and metabolism” (1, 0.46%) was the group with the lowest number of identifiable consensus sequences (Fig. 2a). In the comparison of pre-molt stage and post-molt stage, the cluster of “carbohydrate transport and metabolism” (15, 23.81%) represented the largest group, followed by “amino acid transport and metabolism” (10, 15.87%), “lipid transport and metabolism” (9, 14.29%), “general function prediction only” (9, 14.29%), “Secondary metabolism biosynthesis, transport and catabolism” (8, 12. 7%) and “energy production and conversion” (4, 6.38%), whereas “coenzyme transport and metabolism” (1, 1.59%) was the group with the lowest number of identifiable consensus sequences (Fig. 2b) (Supplementary data 2).

**Figure 3:**
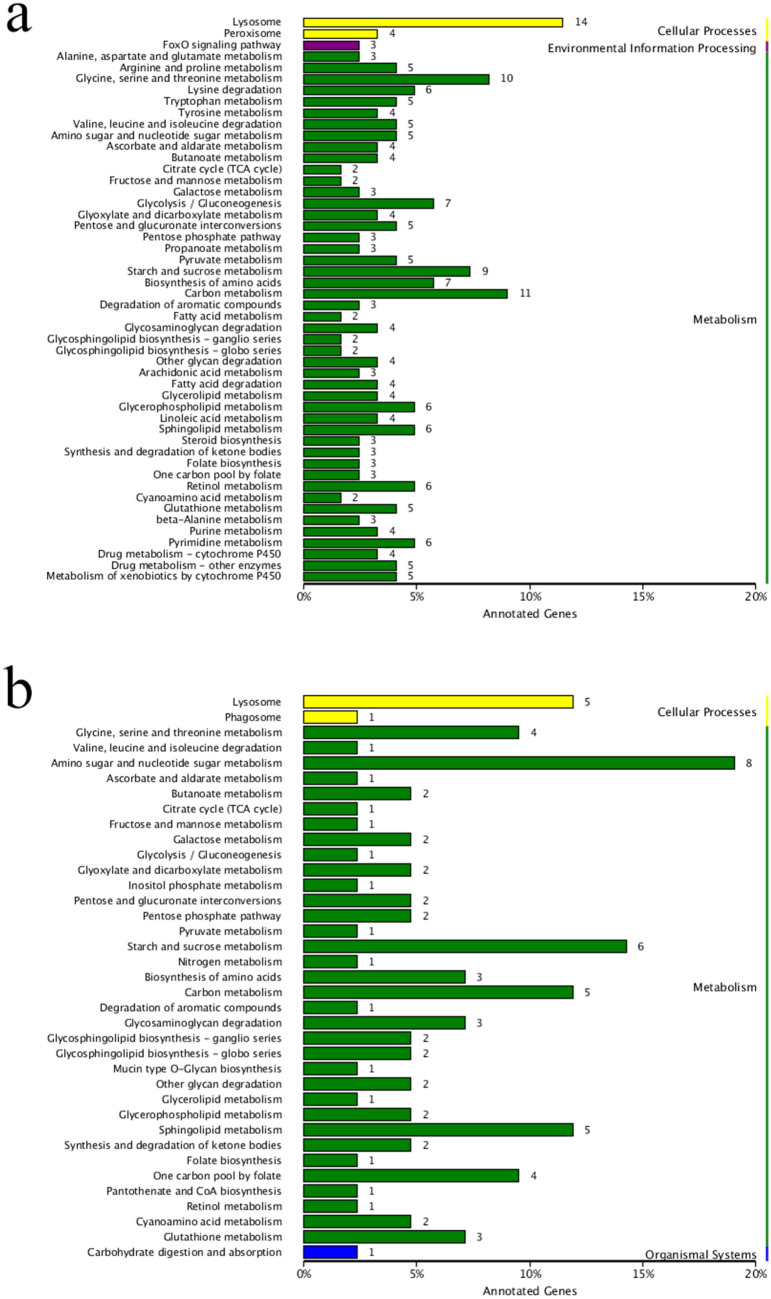
KEGG assignment of non-redundant unigenes for *S. paramamosain* hepatopancreas transcriptome. (**a**) KEGG pathways of the DEGs at inter-molt stage and pre-molt stage. (**b**) KEGG pathways of the DEGs at pre-molt stage and post-molt stage. KEGG assignment is mined here from https://www.kegg.jp/kegg/kegg1.html. The KEGG database has been reported previously^92–94^.

KEGG enrichment results were not obtained from 6 DEGs in the post-molt stage and inter-molt stage comparisons. KEGG enrichment was carried out on 789 DEGs obtained from an inter-molt stage and pre-molt stage comparison, among which 268 DEGs were classified into cellular processes, environmental information processing, metabolism (Fig. 3a). In cellular processes, fourteen genes were assigned to lysosome (ko: 04142) and four genes were assigned to phagosome (ko: 04145); In environmental information processing, one gene related to signal transduction was assigned to FoxO signaling pathway (ko: 04068); In metabolism, carbon metabolism (ko: 01200) and glycine, serine and threonine metabolism (ko: 00260) were the top two pathway (21genes). KEGG enrichment was carried out on 153 DEGs obtained from a pre-molt stage and post-molt stage comparison, among which 67 DEGs were classified into cellular processes, metabolism and organismal systems (Fig. 3b). In cellular processes, five genes were assigned to lysosome (ko: 04142) and one gene was assigned to phagosome (ko: 04145); In metabolism, amino sugar and nucleotide sugar metabolism (ko: 00520) and starch and sucrose metabolism (ko: 00500) were the top two pathways (14 genes); In organismal systems, one gene related to carbohydrate digestion and absorption was identified (ko: 04973) (Supplementary data 3).

### DEGs in the hepatopancreas transcriptome related to immune system

**Figure 4:**
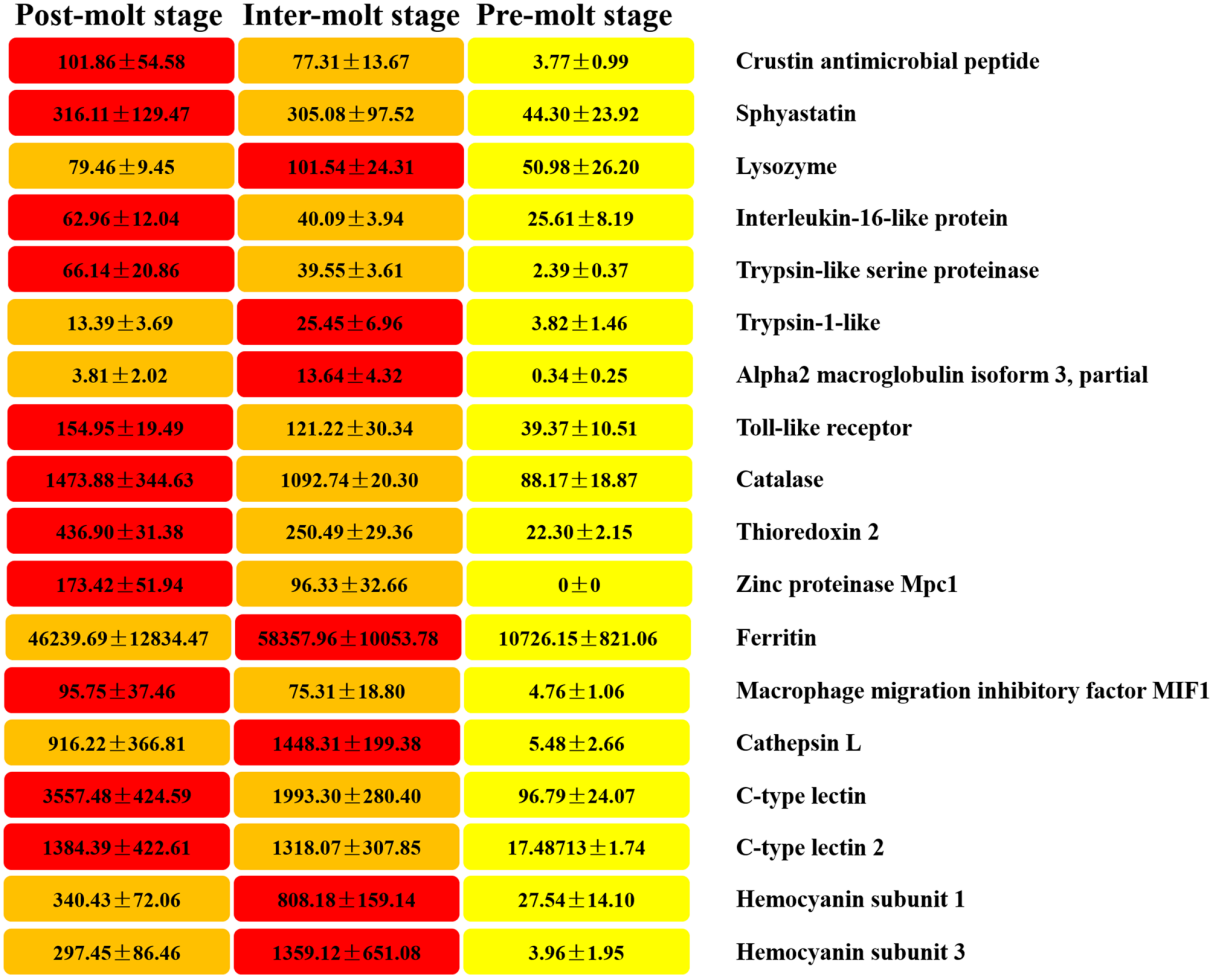
Heatmap showing the abundance of expression of the immune genes. The values in the figure indicate FPKM (fragments per kilobase million). Data are shown as mean ± SEM.

To date, there have been few studies on the immune system in the hepatopancreas of the mud crab. DEGs identified across the different molt stages (post-molt stage, inter-molt stage, and pre-molt stage) were compared pair-wise, and genes related to immune response were screened. Expression abundance of the genes showed a significant difference. Among them, we focused on key genes involved in Toll, immune deficiency (IMD) signaling pathways, immune-related and cytokines (Table 3). We found a large number of highly significant up-regulated immune response genes during the post-molt stage and the inter-molt stage, however, these genes are significantly down regulated and not expressed at the pre-molt stage (Fig. 4) (Supplementary data 4).

**Table 3:** Putative immune genes in the hepatopancreas transcriptome of *S. paramamosain*.

Peptide families	Accession Num	Size(bp)/ (aa)	Best blastx match			
			Species name	E-value	Ident	Accession Num
**Antimicrobial peptides (AMPs)**						
Crustin antimicrobial peptide	c17146.graph_c0	557/185	*Scylla paramamosain*	5e-60	100%	ABY20728.1
Sphyastatin	c34929.graph_c0	742/247	*Scylla paramamosain*	2e-77	100%	AFY10070.1
Lysozyme	c32013.graph_c0	942/314	*Scylla paramamosain*	2e-153	99.55%	ADM33942.1
**Inflammatory gene**						
Interleukin-16-like protein	c54350.graph_c0	6,041/2013	*Eriocheir sinensis*	0.0	90%	AWM96383.1
**Prophenol/phenol oxidase cascade**						
Trypsin-like serine proteinase	c23984.graph_c0	1,011/337	*Eriocheir sinensis*	5e-77	52%	AKN46052.1
Trypsin-1-like	c32762.graph_c0	2,320/773	*Acromyrmex echinatior*	3e-37	36%	XP_011063347.1
Alpha2 macroglobulin isoform 3, partial	c80987.graph_c0	463/154	*Penaeus chinensis*	9e-16	59%	ACU31809.1
**Toll signaling pathway**						
Toll-like receptor	c49018.graph_c0	4,265/1,421	*Procambarus clarkii*	0.0	50.28%	AJE28352.1
**Antioxidant enzymes**						
Catalase	c60548.graph_c0	3,312/1,104	*Scylla paramamosain*	0.0	100%	ACX46120.1
Thioredoxin 2	c29838.graph_c0	1674/558	*Portunus trituberculatus*	5e-72	98%	AFE88627.1
**Metal-binding proteins**						
Zinc proteinase Mpc1	c69961.graph_c0	258/86	*Litopenaeus vannamei*	2e-120	79%	DQ398567.1
Ferritin	c42424.graph_c0	1,251/417	*Scylla paramamosain*	1e-188	99%	ADM26622.1
**Other immune factors**						
Macrophage migration inhibitory factor MIF1	c37813.graph_c0	394/131	*Scylla paramamosain*	7e-73	100%	AKT09427.1
Cathepsin L	c75535.graph_c0	369/123	*Eriocheir sinensis*	3e-40	87%	ADO65978.1
C-type lectin	c10960.graph_c0	712/237	*Eriocheir sinensis*	5e-54	50%	ADH43623.1
C-type lectin 2	c76885.graph_c0	384/128	*Scylla paramamosain*	4e-53	63%	AEO92002.1
Hemocyanin subunit 1	c27342.graph_c0	797/265	*Scylla paramamosain*	8e-140	77%	AKC96433.1
Hemocyanin subunit 3	c94743.graph_c0	226/75	*Scylla paramamosain*	2e-44	99%	AKC96431.1

### Transcript validation by qRT-PCR

**Figure 5:**
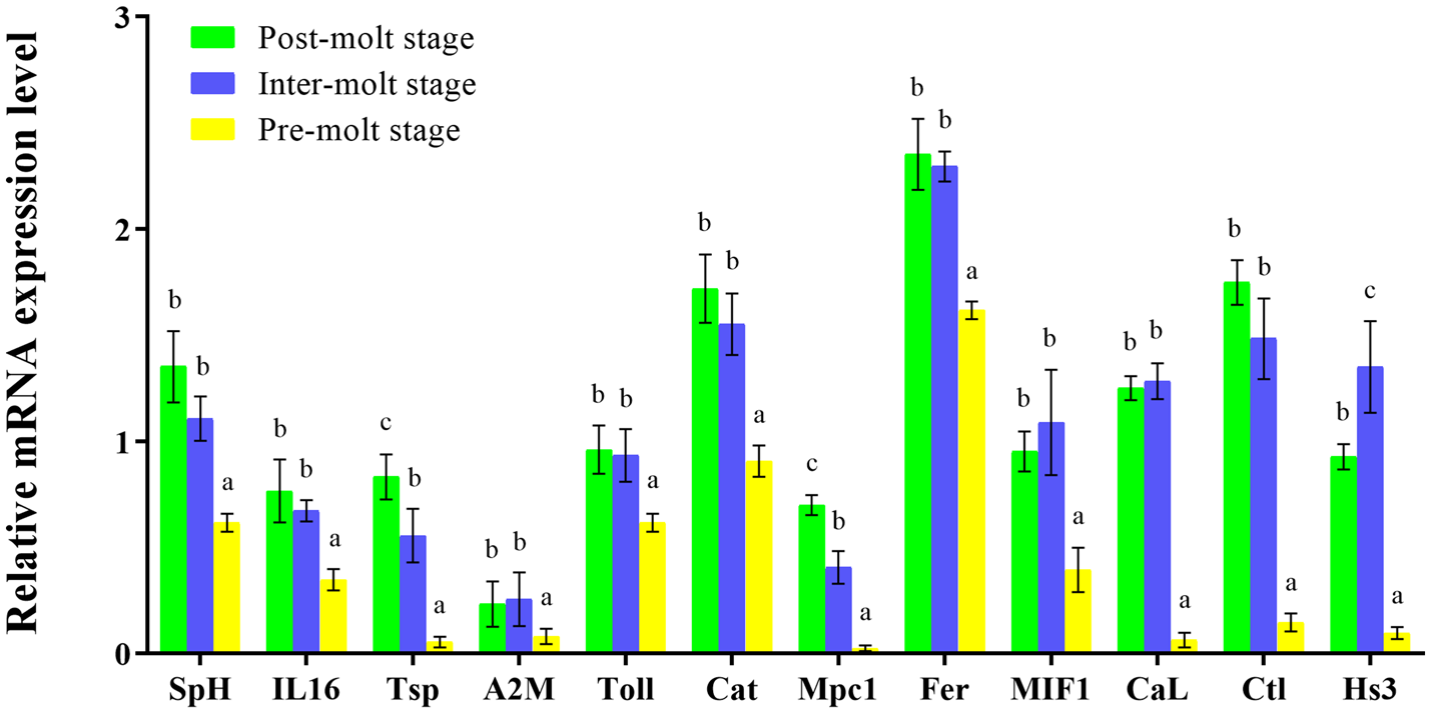
The DEGs of the hepatopancreas at three stages in *S. paramamosain*. *SpH* Sphyastatin, *IL16* Interleukin-16-like protein, *Tsp* Trypsin-like serine proteinase, *A2M* Alpha2 macroglobulin isoform 3, *Toll* Toll-like receptor, *Cat* Catalase, *Mpc1* Zinc proteinase Mpc1, *Fer* Ferritin, *MIF1* Macrophage migration inhibitory factor, *Cal* Cathepsin L, *Ctl* C-type lectin, *Hs3* Hemocyanin subunit 3, *β-actin* was used as reference gene. Columns with different superscript letters indicate the significant difference when compared by ANOVA (P < 0.05).

Twelve immune response genes that identified as differentially expressed genes (DEGs) at post-molt and inter-molt relative to pre-molt were examined by qRT-PCR, and the expression levels of these immune genes were significantly associated with the RNA-seq results (Fig. 5). Our results confirmed the reliability of RNA-seq and accuracy of the Trinity assembly.

## Discussion

In this study, we assembled the hepatopancreas transcriptomes at post-molt, inter-molt and pre-molt stages, and obtained an annotated transcriptome over the molt cycle of the mud crab, *S. paramamosain*. We found classifications of DEGs from GO, COG and KEGG databases, which were involved in energy metabolism, growth development, osmotic pressure regulation and immune-related processes, this phenomenon demonstrated the major physiological changes happen during molt cycle in *S. paramamosain*. Compared with pre-molt stage, a large number of genes were up-regulated at the post-molt stage and the inter-molt stage, which further showed that the hepatopancreas is an important organ involved in molt cycle in *S. paramamosain*. It is interesting to note that some immune-related genes (antimicrobial peptide genes, inflammatory response genes, Toll signaling pathway factors, phenoloxidase system, antioxidant enzymes, metalloproteins, etc.) were significantly up-regulated at the post-molt stage and the inter-molt stage compared with the pre-molt stage, respectively. This might indicate that the mud crabs are sensitive to environmental and physiological changes, and therefore adopt prophylactic immune mechanisms. Similar patterns exist in the life cycle of the swimming crab, *Portunus trituberculatus*, the chitinase gene *PtCht-1* involved in immune defense is highly expressed at post-molt stage and inter-molt stage, but has a low expression at pre-molt stage^21^. It was also reported that the expression of antimicrobial peptides (AMPs) during molt cycle is associated with prophylactic innate immunity in the fruit fly *Drosophila melanogaster*, which acts to prevent infection^22^.

### Antimicrobial proteins (AMPs)

Three unique AMP genes were discovered in the hepatopancreas cDNA library, and they showed a significant upregulation at post-molt stage and inter-molt stage.

Crustins are a cysteine-rich cationic peptide with a characteristic four-disulphide core-containing whey acidic protein (WAP) domain^23^, which has antimicrobial activity against Gram-positive bacteria and no activity against Gram-negative bacteria^24^.

*Sp*Hyastatin was first screened as a new cationic antimicrobial peptide in the suppression subtractive hybridization (SSH) cDNA library constructed from the hemocytes of *S. paramamosain*^25,26^. The previous study has proved that *Sp*Hyastatin can effectively kill microbes via a permeabilizing mechanism and bind to various anionic cell wall components against different species of bacteria^27^.

Lysozymes are immune effectors widely distributed and involved in many physiological processes, such as in immune and digestive systems^28,29^, exerting cytosolic activity on peptidoglycans of bacterial cell walls to initiate cell lysis^30^. In *S. paramamosain*, lysozyme exhibited moderate antimicrobial activity and possessed weak isopeptidase activity and had a strong binding activity to lipopolysaccharide from *Escherichia coli* and lipoteichoic acid and peptidoglycan (PGN) from *Staphylococcus aureus*^30^.

### Inflammatory gene IL-16

As a pro-inflammatory cytokine, Interleukin-16 (IL-16) was first identified as a lymphocyte chemoattractant factor in 1982^31^. IL-16 regulates migration, proliferation and activation of various cells, as well as the release of pro-inflammatory cytokines^32–34^. A recent study revealed that IL-16 might play a vital role in initiating innate immune responses against pathogen infection in *S. paramamosain*^35^.

### Prophenol/phenol oxidase cascade

In the present study, proteases and inhibitors in the phenol oxidase system were significantly up-regulated at the post-molt stage. In crustaceans, the prophenoloxidase activating system (proPO-system) is an important immune defense system. Serine proteases (SPs) constitute one of the largest families of enzymes in the animal kingdom and play important roles in immune responses^36^. In crustaceans, trypsin-like serine proteases have been isolated from the hepatopancreas of the redclaw crayfish, *Cherax quadricarinatus* and the Chinese shrimp, *Fenneropenaeus chinensis*, which are involved in the innate immune defense against pathogens^36,37^. Serine proteinase homologs were also obtained from *S. paramamosain*, it is suggested that *Sp*-SPH protein could bind to a number of bacteria and play a key role in host defense against microbe invasion^38^.

Pacifastin and alpha2-macroglobulin (α2M), ubiquitous protease inhibitors, are involved in the regulation of the prophenyloxidase (proPO) system to avoid the deleterious effects of its active components^39–41^. The expression of these genes is presumed to be due to the activation of the proPO serine proteinase cascade, the over-activated protease is very harmful to cells and tissues, so the expression of serine protease inhibitors is used to control excessive immune responsiveness to maintain homeostasis^39,40^.

### Toll signaling pathway

In this study, the expression of the gene coding for the Toll-like receptor was significantly up-regulated at the post-molt stage and inter-molt stage. The Toll signaling pathway is one of the most important innate immune pathways in invertebrates and has a homologous similarity to the mammalian TLR pathway^41^. Toll/Toll-like receptors (TLRs) were the first pattern recognition receptors (PRRs) identified to play a crucial role in innate immune responses in crustaceans. Up to now, TLRs have been cloned and characterized in a limited number of crustacean species, which suggested that TLRs could regulate the transcripts of several AMPs (*Sp*ALF1-6, *Sp*Crustin, *Sp*Histin, *Sp*Arasin, *Sp*GRP and *Sp*Hyastatin)^42–47^. It is possible that the Toll signaling pathway is activated to regulate the expression of downstream antimicrobial peptides at the post-molt stage, and the expression of antimicrobial peptides was up-regulated in response to different potential pathogens as mentioned above. In addition, we also screened genes related to the Toll pathway, such as Toll-6, dorsal, pelle-like kinase, Myd88 and tumor necrosis factor receptor-associated factor 6 (TRAF6). According to the FPKM data, there are no significant differences at the post-molt stage and inter-molt stage compared with the pre-molt stage, respectively, so they were not listed in Table 3. These genes are the homologs of *Drosophila* Toll pathway genes and involved in Toll-mediated immune defense^48^. It has been reported that the expression profiles of genes involved in the Toll pathway up-regulated at different time points in crustaceans^48–50^.

### Antioxidant enzymes

The present study found that the expression of catalase and thioredoxin were significantly up-regulated after molt, possibly due to the large amount of reactive oxygen species produced by oxidative stress at the post-molt stage. Antioxidant enzymes play essential roles in antioxidant responses caused by pathogen invasion or metabolic process^51^. Catalase is involved in the immune response and plays a role in the protection against oxidative stress in *S. paramamosain*^52^. Thioredoxin, with a redox active disulfide bridge, is responsible for maintaining the balance of reactive oxygen species, which has an important impact on the immune system^53,54^. Thioredoxin has been cloned and characterized in *S. paramamosain* and may prove to be a potential biomarker gene for evaluation of environmental stress in marine species^55^.

### Metal-binding proteins

Two meta-binding proteins were significantly up-regulated at the post-molt stage in *S. paramamosain*. We infer that the tight regulation of ferritin and zinc protease were a primary defense mechanism to resist microbial infection. Metal-binding proteins are involved in many physiological processes, ranging from immune responses to cellular signaling pathways^56^. Ferritin is a metal-chelator protein involved in iron storage, and plays a crucial role in iron metabolism. As some bacteria need to isolate iron, ferritin can protect organisms from bacterial infection by regulating free iron availability in the environment^57^.

Zinc protease belongs to the metal endopeptidases and is involved in a variety of physiological activities, including connective tissue remodeling and resection of nascent protein signal peptides^56^. A full-length ferritin cDNA clone has been isolated from the river prawn, *Macrobrachium nipponense*^58^, however, zinc protease in *S. paramamosain* has not yet been reported. In *L. vannamei* and the kuruma shrimp, *Penaeus japonicas*, mRNA of zinc proteinase and ferritin are more expressed in WSSV resistant individuals compared with susceptible ones^59,60^. These data support the role that these two metal binding proteins may guard against infection events that may accompany cuticle loss and regrowth during the molting process.

### Other immune factors

In the present study, macrophage migration inhibitory factor (MIF), cathepsin L (CTSL), c-type lectins (CTLs) and hemocyanin were significantly up-regulated at the post-molt stage.

MIF is a regulator of the innate immune system, and plays a key role in host antimicrobial defense systems and stress responses^61^. Recently, it was regarded as a multi-functional protein, positioning MIF as a mediator during the inflammatory response to combat infection and in immunoinflammatory and autoimmune diseases^62^. At present, MIF homologs have been reported and studied in many crustacean species, such as *S. paramamosain*^63^, *E. sinensis*^64^, and *Penaeus monodon*^65^*.* In this study, MIF was significantly up-regulated at the post-molt stage, which may indicate that phagocytosis was promoted in *S. paramamosain*.

Cathepsin L (CTSL), a lysosomal cysteine protease, serves as a chemical barrier against microbial invasion in immune responses in vertebrates^66,67^. Cathepsin L has been found in crustaceans, such as *E. sinensis*^69^ and *F. chinensis*^69^, is responsible for lysing pathogenic bacteria and as a barrier against invading pathogens.

C-type lectins (CTLs) are a large superfamily of pattern-recognition receptors that play important roles in the immune system through identifying and binding to the conservative pathogen-associated molecular patterns (PAMPs) on pathogen surfaces^70^. As pattern recognition receptors, CTLs can identify and bind PAMPs on pathogen surfaces to promote the phagocytosis^71,72^. In bivalve and crustacean species, the hepatopancreas synthesizes lectins (such as CTLs and ficolins) in response to infection^11,73,74^.

Hemocyanin, was reported as a novel and important defense molecule of the non-specific innate immune system^75–77^. Studies have found that hemocyanin functions not only as an oxygen-carrying protein, but also as a phenoloxidase-like enzyme^78^, and has antiviral activity, through its enzymatic cleavage, produces AMPs against a variety of viruses^79–81^. Agglutination activities against bacteria have also been observed as an antimicrobial strategy^82,83^.

**Figure 6:**
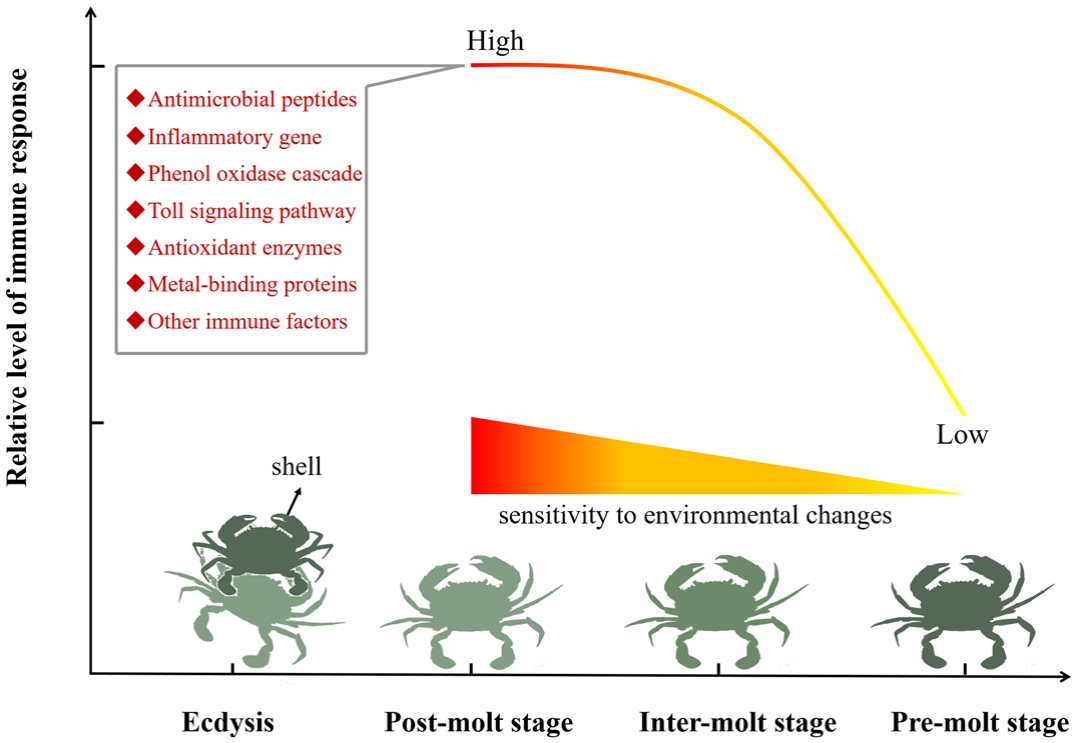
Immune molecular events of *S. paramamosain* during the molting process. This schematic depicts the level of immune response in relation to the environmental sensitivity of the molt cycle. The rhombus represents the molecular events of change; the x-axis represents the stage of molting; the y-axis represents the level of hepatopancreas immune changes.

To our knowledge, this is the first transcriptome analysis of the change of immune related genes in the hepatopancreas during the molt cycle in *S. paramamosain* (Fig. 6). Although these genes are usually multifunctional, they are significantly up-regulated at the post-molt stage and inter-molt stage revealing a potential role of the hepatopancreas in protecting crabs susceptible to infection.

## Materials and methods

### Ethics statement

All animals used in this study have been approved by the Institutional Animal Care and Use Committee of Xiamen University and experimental protocols carried out in strict accordance with the guidelines of the university.

### Sample collection

Mud crabs (*S. paramamosain*), averaging 7.9 ± 0.4 cm in carapace width and 100.2 ± 8.1 g in body weight, were collected from the aquafarm in Xiamen city, Fujian Province, China, and then transported to laboratory. They were reared in tanks (temperature: 26 ± 0.5 °C), fed with shrimp meat, and the water was changed every day. Mud crabs at three molt stages were sampled according to the previous study^16^. Post-molt stage: the mud crab just molted for 1–2 days, it was very thin, only dorsal carapace edge hard,Inter-molt stage: the dominant phase of the inter-molt period, the mud crab was full of meat, the exoskeleton was consolidated and hardened,Pre-molt stage: the fourth phase of the pre-molt period, namely double shell crab, pleural suture cracks between the carapace and the abdomen. Three individual crabs at each molt stage were sampled, placed on ice for anesthetization and then dissected for the hepatopancreas for RNA isolation. Three biological replicates were performed per tissue at each molt stage, for a total of 9 samples.

### RNA isolation and cDNA library preparation

Total RNA was extracted from each tissue using TRIzol RNA isolation reagent (Invitrogen) according to the manufacturer’s instructions. The RNA quantity and integrity were determined with an Aglient 2100 Bioanalyzer and a Qubit 2.0 Fluorometer. Magnetic beads with oligo (dT) were used to separate Poly-(A)-containing mRNA. Fragmentation buffer was added to randomly interrupt the mRNA into short fragments. The mRNA fragments were used as templates to synthesize the first-strand cDNA with random hexamer-primers, then add buffer, dNTPs, RNase H, and DNA polymerase I to synthesize the second-stranded cDNA, and further purified using Agencourt AMPure XP beads (Beckman Coulter). Next, double-stranded cDNA fragment ends were repaired, adding of poly (A) and ligation of adapters. The fragment size was selected using Agencourt AMPure XP beads. The PCR amplification to create the final cDNA library, and sequenced using the Illumina HiSeq2500.

### Cleaning and de novo assembly of sequencing reads

The assembles to compiled data from all 9 samples were assembled together for assembly, as this method is often more accurate and comprehensive for samples from the same species without a genome-wide reference^84^. The Trinity software first breaks the sequencing Reads into shorter fragments (K-mer), then extends the small fragments into longer fragments (Contig), and uses the overlap between the fragments to get the fragment. Finally, using the De Bruijn map method and sequencing Read information, the transcript sequences are separately identified in each fragment set^85^. The resulting clean readings were archived at the National Center for Biotechnology Information (NCBI) Sequence Read Archive, accession number is PRJNA617953.

### Transcriptome functional annotation

Assembled transcriptomes were annotated using BLASTX against the Swiss-Prot, GO, NCBI-NR, COG, KEGG, with a cutoff E-value smaller than 1e-5. Analysis of Unigene results in KEGG Orthology using KOBAS 2.0 software^86^. HMMER software compares the predicted amino acid sequence with the Pfam database to obtain Unigene annotation information, with a cut off E-value smaller than 1e−10. TransDecoder software is based on the open reading frame (ORF), the alignment of the amino acid sequence with protein domain sequence in Pfam (Protein family) database and other information, which can identify reliable potential coding sequence (CDS) from the transcript sequences. The Reads sequenced from each sample were mapped back to Unigene database using Bowtie^87^, and the expression level was estimated according to the comparison results combined with RSEM^88^ (RNA-Seq by Expectation Maximization). The fragments per kilobase of transcript, per million fragments sequenced (FPKM) value indicates the expression abundance corresponding to each unigene.

### Differential expression analysis

Pearson's Correlation Coefficient (r) serves as an evaluation index of biological repeat correlation^89^. DESeq^90^ was used to identify the differentially expressed genes (DEGs). To ensure high quality of DEGs, Benjamini-Hochberg-corrected *P* values (*q* values) were calculated for one multiple hypothesis testing false discovery rate (FDR < 0.01) and fold change (FC ≤ 2) adopted as the key indicator for DEGs. The expression levels of up-regulated or down-regulated DEGs were annotated in the GO annotation database were enrichment analyzed using the topGO software^91^. COG database can perform orthologous classification of gene products and KEGG database helps to further interpret the function of gene. The Enrichment Factor was used to analyze the enrichment of pathway, and the Fisher's exact test method was used to calculate the enrichment significance.

### Gene expression validation

To validate and quantify the transcriptome data, genes identified in the DEGs were selected and then quantified using quantitative real-time PCR (qRT-PCR). Primers were designed using Primer 5.0 Tool (Premier, Canada). The stable housekeeping gene *β-actin* was used as an internal control (Table 4). qRT-PCR using a 7,500 Fast Real-Time PCR system (Applied Biosystems) was performed with the following thermal profile: One cycle at 95 °C for 30 s, followed by 40 cycles of 95 °C for 5 s, 58 °C for 30 s, and 72 °C for 30 s. The qRT-PCR was carried out in triplicate for each sample, and five mud crabs were analyzed in each group. Expression levels of different genes were estimated using the 2^–ΔΔCt^ method. Statistical significance (P < 0.05) was determined using Duncan’s multiple range tests and one-way ANOVA under SPSS 18.0.

**Table 4:** The primers used in qRT-PCR.

Name	Sequence (5′ → 3′)
*SpH-F*	GCACCAAGCCCTTGATCTCC
*SpH-R*	GTTGCTGCGGGTGAAGGAAT
*IL16-F*	CTCGCAGGGAAAGCAATGGT
*IL16-R*	GGCTGTTGGACGAAGACTGG
*Tsp-F*	CCCTTCTGTTGGCAGTGCTT
*Tsp-R*	GCTTGGTCTGCTCCACAGTC
*A2M-F*	GAGGCTGGTAAGGGCTGACT
*A2M-R*	GAGAATGTGTGCGTGCGGAT
*Toll-R*	GCTCCAGGAAGGTGTTGGTG
*Toll-F*	AGGAAGCGCAGTTTCTTGGG
*Cat-F*	TGGGAGCCAACTACCACCAA
*Cat-R*	GCAGTCCATAGGGCCAGAGA
*Mpc1-F*	GGCACGTACTCCTTCTCCGT
*Mpc1-R*	TGTGTGCAGTTTCGTGTTGC
*Fer-F*	GCCCTACCACGCCATTGATT
*Fer-R*	GATGGAGGCCTCACACTCCT
*MIF1-F*	CCTGTGTGTGAGCTGCATGT
*MIF1-R*	GGCTGCAAGGCTGAGAAGTT
*CaL-F*	GTACCGTAACCCACCGCAAG
*CaL-R*	TCCCAGCCCACTTTCCACTT
*Ctl-F*	GTCTGTGGCAAATGCAGGGT
*Ctl-R*	AGATTCCCGTCGTGACCACT
*Hs3-F*	CGACCCACATGGCAAGTTCA
*Hs3-R*	CGTGTAGCGGTCTCGAAGTG
*β-actin-F*	GAGCGAGAAATCGTTCGTGAC
*β-actin-R*	GGAAGGAAGGCTGGAAGAGAG

## Supplementary information

Supplementary data1.

Supplementary data2.

Supplementary data3.

Supplementary data4.
